# Smart Electronic Textiles for Wearable Sensing and Display

**DOI:** 10.3390/bios12040222

**Published:** 2022-04-08

**Authors:** Seungse Cho, Taehoo Chang, Tianhao Yu, Chi Hwan Lee

**Affiliations:** 1 Weldon School of Biomedical Engineering, Purdue University, West Lafayette, IN 47907, USA; cho475@purdue.edu; 2 School of Materials Engineering, Purdue University, West Lafayette, IN 47907, USA; chang466@purdue.edu; 3 School of Mechanical Engineering, Purdue University, West Lafayette, IN 47907, USA; yuth@purdue.edu; 4 Center for Implantable Devices, Purdue University, West Lafayette, IN 47907, USA

**Keywords:** electronic textiles, wearable sensing and display, smart clothing, textile engineering, ambulatory health monitoring

## Abstract

Increasing demand of using everyday clothing in wearable sensing and display has synergistically advanced the field of electronic textiles, or e-textiles. A variety of types of e-textiles have been formed into stretchy fabrics in a manner that can maintain their intrinsic properties of stretchability, breathability, and wearability to fit comfortably across different sizes and shapes of the human body. These unique features have been leveraged to ensure accuracy in capturing physical, chemical, and electrophysiological signals from the skin under ambulatory conditions, while also displaying the sensing data or other immediate information in daily life. Here, we review the emerging trends and recent advances in e-textiles in wearable sensing and display, with a focus on their materials, constructions, and implementations. We also describe perspectives on the remaining challenges of e-textiles to guide future research directions toward wider adoption in practice.

## 1. Introduction

According to a new market research report published by Research and Markets, the global market for wearable technology is anticipated to grow and reach > $118.16 billion by 2028 [1]. The most compelling version of wearable products demands prolonged contact to the skin, enabling the high-precision detection of physical, chemical, and electrophysiological signals associated with chronic health conditions or infectious diseases, particularly in pandemic circumstances [2,3,4,5,6]. In this field, an overarching challenge is that the rigid or semi-flexible form of electronics is unable to intimately interface with the soft, irregular surface of the skin [7,8,9]. This mechanical mismatch causes discomfort to the wearer as well as a low signal-to-noise ratio (SNR) in collecting data.

Among many wearable products, e-textiles in stretchy fabrics have been of great interest in both academic and industrial fields due to their ultimate wearability to fit seamlessly across different sizes and shapes of the human body [10,11,12,13,14]. Over the past decade, various types of e-textiles have been developed not only to capture physical, chemical, and electrophysiological signals from the skin under ambulatory conditions but also to visualize the sensing data or other immediate information in daily life [15,16,17,18]. Several e-textiles have been commercialized in healthcare and sport markets in the form of smart socks, shirts, bras, sleeves, and gloves for the wearable sensing of walking speed, blood pressure, skin temperature, respiration rate, cardiac rhythm, blood oxygen level, and daily activity level [13]. In parallel, e-textiles that incorporate photometric or colorimetric units have been also used in wearable displays not only for fashion illuminating apparel but also as communication tools, visual aids, safety precautions, and health monitoring [18,19,20]. The wearable sensing and display can be also integrated into a closed loop circuit for the real-time display of the sensing data.

E-textiles have typically been formed by weaving, knitting, or embroidering functional fibers into fabrics in a coaxial or twisted layout or directly embedding functional nanoparticles into fabrics [21,22,23]. The different kinds of methodologies have their own advantages and disadvantages [24]. The weaving of textiles can facilitate the fabrication of multilayers that can hide conductive yarns in a woven textile to prevent short circuits, but the resulting woven structures need a predetermined location of weft or warp yarns, which restricts the fabrication freedom. Knitted textiles are relatively stretchable but require notably higher conductivity of transmission lines than yarns in the sensing area to accommodate the deformation of yarn loops. The embroidery of textiles has high freedom in placing conductive lines but induces a high level of stress to yarns during the embroidery process, thereby limiting the choice of yarns. There are several key requirements to maintain the intrinsic fabric features including (1) breathability for user comfort; (2) conformability to the irregular surface of the skin without irritating wearers; (3) deformability against natural body movements (i.e., motion artifacts) including stretching, bending, and twisting without degrading the measurement accuracy and reliability of e-textiles; (4) durability against multiple reuses and laundry cycles; and (5) biocompatibility for long-term use on the skin without noticeable side effects. All these features are critically important for the pragmatic implementation of e-textiles into practice.

**Figure 1:**
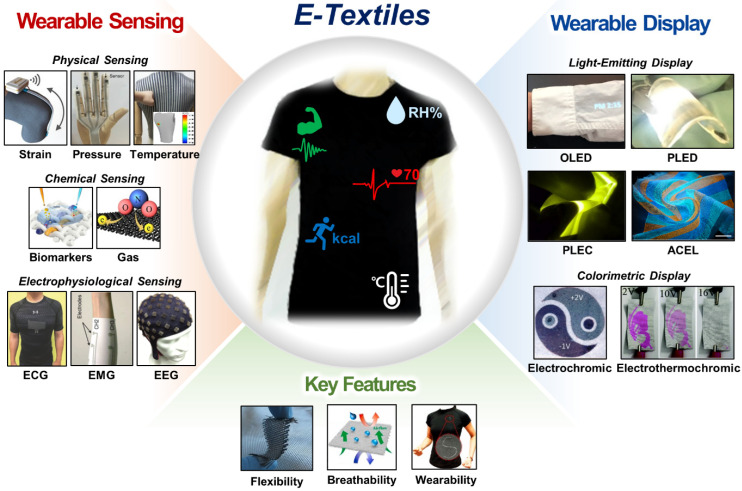
E-textiles for wearable sensing and display. Physical sensing—strain: reprinted with permission from [25]. Copyright 2015 American Chemical Society. Pressure: reprinted with permission from [26]. Copyright 2017 Wiley-VCH. Temperature: reprinted with permission from [27]. Copyright 2020 American Chemical Society. Chemical sensing—biomarkers: reprinted with permission from [28]. Copyright 2020 Springer Nature. Gas: reprinted with permission from [29]. Copyright 2021 American Chemical Society. Electrophysiological sensing—electrocardiogram (ECG): reprinted with permission from [30]. Copyright 2018 Wiley-VCH. electromyogram (EMG): reprinted with permission from [31]. Copyright 2017 Wiley-VCH. Electroencephalogram (EEG): reprinted with permission from [32]. Copyright 2015 Springer Nature. Light-emitting device (LED)—organic light-emitting diode (OLED): reprinted with permission from [33]. Copyright 2020 Springer Nature. Polymer light-emitting diode (PLED): reprinted with permission from [34]. Copyright 2016 Elsevier B.V. Polymer light-emitting electrochemical cell (PLEC): reprinted with permission from [35]. Copyright 2016 IOP Publishing. Alternating current (AC)-driven electroluminescent device (ACEL): reprinted with permission from [36]. Copyright 2021 Springer Nature. Colorimetric device—electrochromic: reprinted with permission from [37]. Copyright 2021 Wiley-VCH. Electro-thermochromic: reprinted with permission from [38]. Copyright 2020 Royal Society of Chemistry. Key Features—flexibility: reprinted with permission from [39]. Copyright 2011 Wiley-VCH. Breathability: reprinted with permission from [40]. Copyright 2021 Elsevier B.V. Wearability: reprinted with permission from [41]. Copyright 2018 American Chemical Society.

Herein, we review the latest advances, mainstream ideas, and current limitations of e-textiles in wearable sensing and display. In each section, we discuss the active materials, basic configurations, fabrication methods, and key features of e-textiles with an outline according to their applications in (1) physical sensing, (2) chemical sensing, (3) electrophysiological sensing, and (4) wearable display (Figure 1). We also describe the unmet challenges and future research directions of e-textiles to motivate further advances towards their wide adoption in practice.

## 2. Physical Sensing (Temperature, Strain, and Pressure)

Any unusual change in skin temperature or hyperthermia could be a sign of abnormal health conditions, such as internal bleeding, infection, heat stroke, adverse events, and mortality [42,43,44,45,46]. Thus, several e-textiles have been utilized by relying on either resistive or thermoelectric sensing of temperature on the skin or from the surroundings. The resistive sensing typically utilizes metallic wires (e.g., Au, Pt) or carbon-based nanomaterials (e.g., carbon nanotube (CNT), reduced graphene oxide (rGO)) in which their change in resistance by temperature is defined as $R_{T}=R_{\mathrm{ref}}\;\left[1+\alpha_{\mathrm{ref}}\left(T-T_{\mathrm{ref}}\right)\right]$, where *R*_T_ and *R*_ref_ are the resistance of temperature sensitive electrode and reference electrode; *T* is the effect of temperature; *T*_ref_ is the reference temperature; and *α*_ref_ is the temperature coefficient of resistivity for a given temperature range, respectively [47]. The temperature coefficient of resistance (TCR) is defined as $\Delta R/R_{0}\Delta T$. The thermoelectric sensing utilizes Seebeck coefficient materials, such as poly(3,4-ethylenedioxythiophene):poly(styrene sulfonate) (PEDOT:PSS), that can produce an electric potential by temperature differences, which is defined as ΔV=−SΔT, where Δ*V* is the voltage change; *S* is the Seebeck coefficient; and Δ*T* is the temperature change, respectively [27,48].

Apart from temperature, the continuous and unobtrusive monitoring of strain/pressure from a small (e.g., vibration of the vocal cords/increase of cranial pressure) to large (e.g., abdominal respiration, joint movements/weight pressure on the foot) range of physical activities is also critical to understand the underlying biomechanics of muscular responses in robotics, prosthetics, and human–machine interfaces [49]. Either resistive, capacitive, or triboelectric sensing has been utilized. The resistive sensing utilizes Ag nanowires (Ag NWs) [50], Ag nanoparticles (Ag NPs) [51], poly(3,4-ethylenedioxythiophene) (PEDOT) [52], polypyrrole [53,54,55], CNTs [56,57,58], rGO [59,60], and/or carbon black [61], in which their change in resistance by applied strain/pressure is defined as $\Delta R/R_{0}=G\varepsilon$, where Δ*R* is the resistance change; *R*_0_ is the initial resistance; *G* is the gauge factor; and *ε* is the applied strain, respectively [62]. The capacitive sensing utilizes dielectric materials (e.g., polydimethylsiloxane (PDMS) [63], Ecoflex [64], ion-gel [65], nitrile rubber [66]) with metallic electrodes in which their capacitance is given by $C=\varepsilon_{0}\varepsilon_{r}\left(A/d\right)$, where *ε*_0_ is the space permittivity; *ε*_r_ is the relative dielectric constant of the dielectric material; *A* is the area of the capacitor; and *d* is the distance between separated electrodes, respectively [63]. The triboelectric sensing is based on the contact–separation between two different frictional materials with opposite triboelectric polarities (e.g., nylon/conductive yarns [67,68]) to generate the output voltage (i.e., open-circuit voltage), which is described as $V_{P}=\sigma_{0}d_{P}/\varepsilon_{0}$, where *σ*_0_ is the triboelectric charge density; *d*_P_ is the gap distance under pressure; and *ε*_0_ is the space permittivity, respectively [69]. In the following sections, a number of e-textiles are reviewed in their use for the continuous and unobtrusive monitoring of temperature, strain, and pressure.

### 2.1. Temperature Sensing

**Figure 2:**
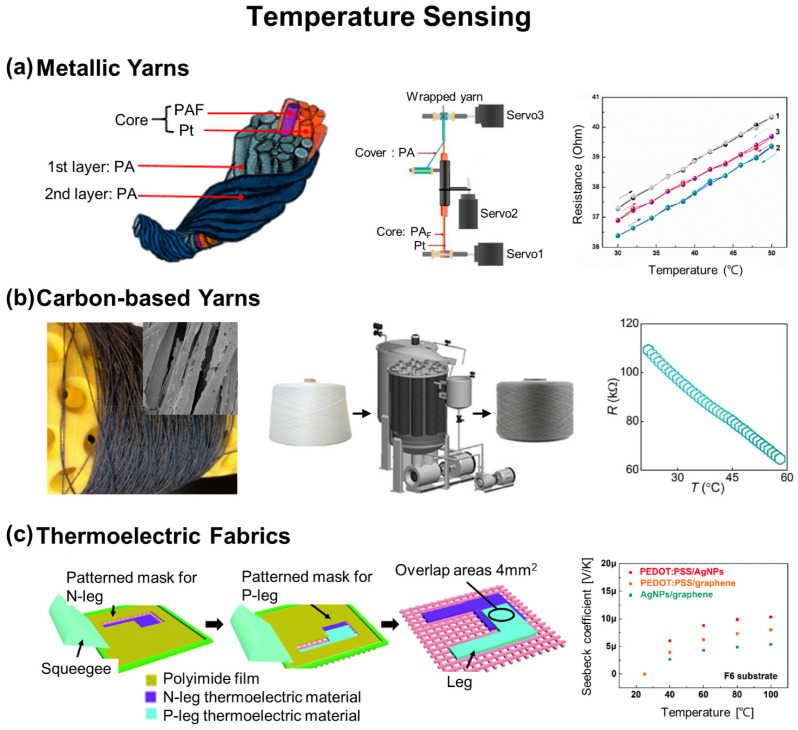
E-textiles for temperature sensing. (**a**) Metallic yarn-based sensing. Reprinted with permission from [70]. Copyright 2019 MDPI. (**b**) Carbon-based yarn-based sensing. Reprinted with permission from [72]. Copyright 2019 American Chemical Society. (**c**) Thermoelectric fabric-based sensing. Reprinted with permission from [48]. Copyright 2018 Royal Society of Chemistry.

Figure 2a shows an example of a temperature sensing e-textile made of a Pt fiber wrapped by two polyamide 66 fibers using hollow-spindle spinning [70]. After the wrapping, the breaking force of the Pt fiber at a fixed diameter of 20 µm was dramatically increased from 17.79 (±0.56) to 47.36 (±2.39) N at its electrical failure. The Pt fiber was mechanically robust sufficiently to be woven into cottons to form a temperature sensor that exhibited a sensitivity of 0.00358 °C^−1^ within a range of 30–50 °C. To increase the sensitivity up to a comparable level of a commercial Pt1k temperature sensor (~0.25 Ω °C^−1^), stainless steel microwires (AISI 304) wrapped with polyester threads were alternatively used by embroidering them in a helical meander-shaped structure on synthetic fabrics [71].

Figure 2b shows a temperature-sensing e-textile with the addition of an overcoat of rGO flakes on a bleached cotton yarn by using a batch-dyeing machine in a high-throughput manner (1000 kg h^−1^) [72]. After the overcoat, the cotton yarn was knitted into an interlocked scaffold shape using an automatic knitting machine, which provided a high mechanical reliability against a cyclic test at 25–55 °C. Other than rGO flakes, carbon-based nanomaterials, such as CNTs, have been also used due to their high sensitivity and fast response time in temperature sensing [73]. For instance, Hasanpour et al. deposited CNTs and fluorinated ethylene propylene (FEP) on cotton threads by multiple dip-coating and drying processes as an insulator to prevent the effect of humidity on temperature sensing [74]. The threads were then stitched into polyester fabrics using a standard stitching machine, which exhibited a sensitivity of −0.31% °C^−1^ within a range of 50–120 °C without a significant hysteresis in cyclic testing. To further increase the sensitivity up to the current highest level of 23.3 kΩ °C^−1^ (or 1.23% °C^−1^) among other fibrous temperature sensors, Wu et al. invented a hybrid structure of silk fiber by mixing CNTs in an ionic liquid of 1-ethyl-3-methylimidazolium bis(trifuloromethylsulfonyl)imide to provide additional charge transport paths [50]. The hybrid structure was effective in enhancing the sensitivity due to the combination of the percolation theory of CNT networks and fast ionic mobility of ionic liquid in response to temperature changes.

Figure 2c shows a temperature sensing e-textile by exploiting a thermoelectric effect which can be also used as a self-powered system [48]. Specific patterns of Ag NPs and PEDOT:PSS were subsequently deposited on fabrics using shadow masks to define n-type and p-type thermoelectric elements, respectively. The combination of n-type Ag NPs and p-type PEDOT:PSS produced an output Seebeck voltage of 1.1 mV at a temperature gradient of 100 K with linear responses in a range of 30–100 °C. The thermoelectric properties of the e-textile decreased only 7% after 800 cycles of stretching at the applied strain of 20%, which is beyond the limit of inorganic thermoelectric materials such as Bi, Te, and Sb. A 5 × 5 array of sensing across an area of 25 mm^2^ was demonstrated to spatially map external heat stimuli in real time. 

### 2.2. Strain and Pressure Sensing

**Figure 3:**
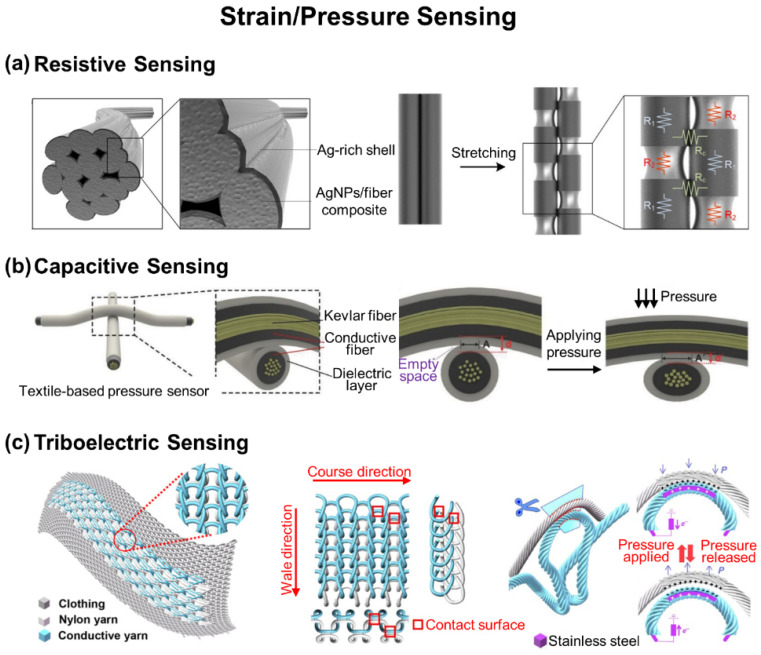
E-textiles for strain and pressure sensing. (**a**) Resistive sensing with an Ag NP/fiber composite/Ag-rich shell fiber. Reprinted with permission from [75]. Copyright 2018 American Chemical Society. (**b**) Capacitive sensing with an Ag NP/SBS/Kevlar fiber. Reprinted with permission from [63]. Copyright 2015 Wiley-VCH. (**c**) Triboelectric sensing with a Terylene-twisted stainless-steel/nylon yarn. Reprinted with permission from [68]. Copyright 2020 American Association for the Advancement of Science.

Figure 3a shows an example of a strain-sensing e-textile made through an Ag NPs-rich shell-formation method on a stretchable polyurethane (PU) fiber [75]. Specifically, an Ag precursor (i.e., AgCF_3_COO) was absorbed in a PU fiber by immersing in a mixture solution of hydrazine hydrate and ethanol for 30 min to reduce Ag^+^ ions into Ag NPs. Following repetitive absorption and reduction steps of the Ag precursor, the resulting PU fiber had inner Ag NP/fiber composite and outer Ag-rich shell with an electrical conductivity of 20,964 S cm^−1^. The PU fiber provided a high gauge factor of 35 and 659 at a strain of 100% and 150–200% with a wide sensing range of up to 450%, respectively. The high sensing performance is attributed to the independent structural deformation of outer Ag-rich shell, which cracked first when external strain was applied at a low strain range (<150%) and then inner Ag NP/fiber composite at a high strain level (>150%). The e-textile was formed by sewing sensory fibers into the finger nodes of a glove to control a robotic hand for human–machine interfaces, showing a distinguishable response to the bending of fingers without interferences between each sensory fiber. In addition, an artificial bladder system was also demonstrated using a sensory fiber to monitor the volumetric change of a pig bladder and showed stable resistive responses with regard to the multiple injection and extraction of external liquids, verifying its in vivo applicability.

Figure 3b shows a capacitive sensing e-textile using an Ag NPs/poly(styrene-block-butadienstyrene) (SBS)-coated Kevlar fiber along with a PDMS dielectric shell [63]. A 30 cm-long Kevlar fiber was vertically fixed on a stand for the drop-casting of SBS solution along the surface with a flow rate of 2 mL s^−1^. Following a drying step at room temperature for 5 min, the SBS-coated Kevlar fiber was immersed into a solution of AgCF_3_COO for 30 min to absorb Ag^+^ ions into the fiber. A mixture solution of hydrated ethanol and water was then drop-casted to reduce the Ag^+^ ions into Ag NPs. The resulting Kevlar fiber showed an excellent electrical resistance of 0.15 Ω cm^−1^ with a high stability against 3000 cycles of bending. The Kevlar fiber was integrated with gloves and cloths for their use in human-machine interfaces to wirelessly control a flying drone and Hexapod walking robot, which exhibited a high sensitivity of 0.21 kPa^−1^ at a pressure range under 2 kPa within a millisecond range of response time. As a notable example, Tabor et al. developed a 3 × 3 array of capacitive sensing e-textile through a scalable sewing of commercial conductive and insulating yarns that are made of Ag-plated polyamide 66 and styrene-ethylene-butylene-styrene (SEBS), respectively [76]. The e-textile was used to map the distribution of pressure across the surface of a limb with respect to its tilting angle ranging from −15° to +9°, which could also be useful in continuously monitoring pressure around the surface of a prosthetic socket to help prevent ulcers.

Figure 3c shows a triboelectric sensing e-textile using a conductive Terylene-twisted stainless-steel yarn that is interlocked with a nylon yarn by knitting in a full cardigan stitch [68]. When the two different yarns were in contact with each other with an applied pressure, equivalent charges of opposite polarities could be generated across their surface by triboelectrification. When they were separated, positive charges could be induced in the inner stainless-steel electrode by electrostatic induction, thereby generating electricity. The e-textile exhibited a high sensitivity of 7.84 mV Pa^−1^; fast response time of 20 ms; and wide working frequency bandwidth up to 20 Hz, which allowed it to detect pulse waveforms from the neck, fingertip, and ankle as well as respiration rates from the chest. The e-textile was mechanically robust as a washable fabric over 40 times. As a notable example, Meng et al. demonstrated the utility of a triboelectric sensing e-textile for the self-powered monitoring of health [67]. The e-textile was able to detect the pressure from blood vessels during the contact-separation between a single three-ply twisted polyester-metal hybrid fiber and an Ag-coated fabric. With an external force, the triboelectrification-induced charges on the surface of the metal fiber were transferred to the Ag-coated fabric by electrostatic induction, resulting in triboelectric electrical signals. The e-textile exhibited an increased sensitivity of 3.88 mV Pa^−1^ at 0.1–4.3 kPa, which allowed it to detect pulse waves from the wrist for the diagnosis of obstructive sleep apnea–hypopnea syndrome from a distance via a smartphone.

## 3. Chemical Sensing

Immediate detection of biological analytes and hazardous chemicals from surroundings is critically important for preventative healthcare in many workplaces [77]. In this context, e-textiles could be beneficial to wearers for the prompt notification of health or environmental conditions by continuously detecting various biomarkers or toxic chemicals in a wearable fashion. A variety of types of e-textiles have been developed for the detection of biomarkers (e.g., glucose [78,79,80,81,82], lactate [80,83,84], pH [28,85], hemoglobin [79], ions [28,86]) from sweat, which can be generally grouped into two categories, using either (1) electrochemical sensing (e.g., amperometry, potentiometry, voltammetry) or (2) organic electrochemical transistors (OECTs) sensing. For electrochemical sensing, amperometry is a simple way to measure an electrical current generated from a redox reaction between electroactive species at a constant potential, which can provide simple detection and easy post processing to convert current to the concentration of target analyte. Potentiometry and voltammetry are techniques to measure any change in the concentration of target ions without interference from other charged ions and a current response of a redox active solution to cyclic potential sweeps between sensing and reference electrodes, respectively. For OECTs sensing, the sensors are used to detect the change of electrical current or potential at a channel or gate in response to electrochemical reactions with a low power consumption at an operational potential of <1 V, which makes it suitable for portable and wearable devices. The e-textiles for OECTs sensing have been typically formed by combining both metallic and PEDOT:PSS yarns because of their high electrical conductivity and stability, enabling the detection of various biomarkers, such as tyrosine and electrolytes [86,87,88]. Sample collection is an important issue in chemical sensing. Hydrogel-based patches have been utilized for profiling skin metabolites [89]. Microfluidics have been also utilized for achieving controlled transport of biofluids through a chemical treatment or photolithography on the surface [90,91]. Nonetheless, these approaches remain challenging to directly integrate with e-textiles due to either the absorption property of fabrics or their potential contamination after each use. Recently, a Janus-wettable textile was developed to facilitate the unidirectional sweat extraction from skin to e-textiles, which can potentially overcome the challenge of sample collection [92].

Continuous monitoring of toxic and hazardous chemical gases, such as ammonia (NH_3_), nitrogen dioxide (NO_2_), hydrogen (H_2_), hydrogen peroxide (H_2_O_2_), hydrogen sulfide (H_2_S), carbon dioxide (CO_2_), and sulfur dioxide (SO_2_), from surroundings is also of great interest for safety and health at work. For instance, NO_2_ is highly toxic, NH_3_ is corrosive, and H_2_ is flammable, all of which are harmful to human health and therefore require an immediate detection. A variety types of e-textiles have been developed to detect gases by typically relying on chemiresistive sensing with graphene derivatives [29,93,94,95,96,97,98,99,100,101], CNTs [102,103], polyaniline [104,105], and metal–organic semiconductors [106]. However, the repeatability of e-textiles in gas sensing still needs to be improved as their performance tends to dramatically degrade after several sensing cycles due to the incomplete adhesion and separation of chemicals from conducting materials.

Securing the biosafety of the constituent materials in these e-textiles is critically important to prevent any harmful responses to the skin. In particular, when Ag presents in the form of nanoparticles instead of bulk, it may cause skin irritation due to the increased release of Ag ions by the change in physico-chemical properties at the nanoscale [107]. Recently, biocompatible materials with the combination of carbon black and natural polymers (e.g., coconut oil, chitosan) have been pursued to improve biocompatibility in wearable sensors [108,109].

In the following sections, a number of e-textiles are reviewed in their use for the accurate monitoring of biomarkers and gases.

### 3.1. Biomarker Sensing

**Figure 4:**
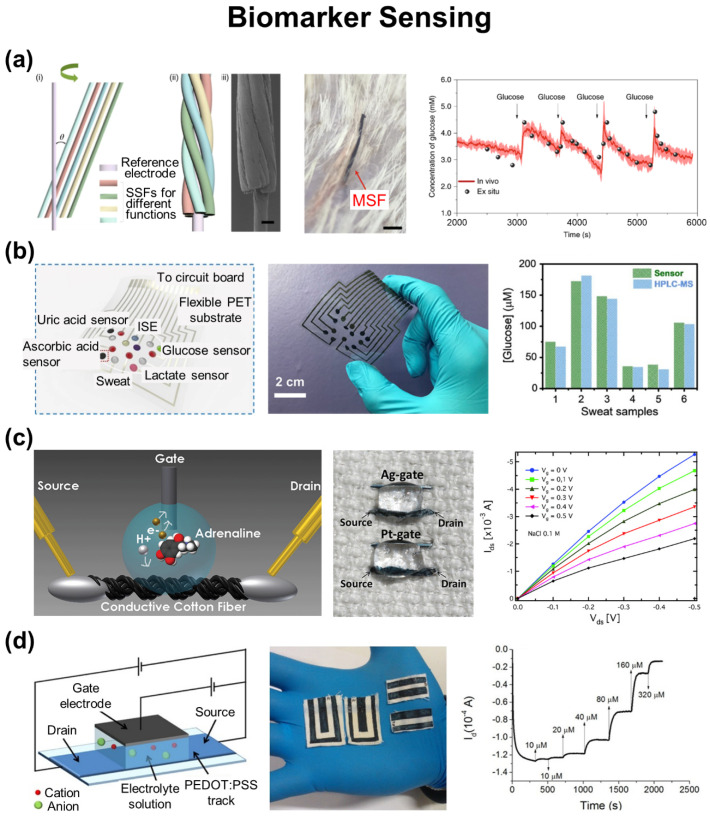
E-textiles for biomarker sensing. (**a**) Multi-ply electrochemical sensing fiber (MSF) with helical CNT bundles. Reprinted with permission from [110]. Copyright 2019 Springer Nature. (**b**) Electrochemical SilkNCT-based wearable sweat analysis patch. Reprinted with permission from [112]. Copyright 2019 American Association for the Advancement of Science. (**c**) OECTs fiber with PEDOT:PSS. Reprinted with permission from [113]. Copyright 2014 Royal Society of Chemistry. (**d**) OECTs fabric with PEDOT:PSS. Reprinted with permission from [114]. Copyright 2016 Springer Nature.

Figure 4a shows an electrochemical sensing e-textile using CNT bundles as a core electrode that can be coated with a number of active elements according to target analytes [110]. For instance, the CNT bundles were coated with Pt nanoparticles for the chronoamperometric detection of H_2_O_2_ through their catalytic dissociation, yielding the current response, sensitivity, and linear detection range of 0–50 µM, 0.84 µA µM^−1^, and 0–1.0 mM, respectively. The CNT bundles were coated with chitosan-Pb_2_[Fe(CN)_6_]-poly(diallyldimethylammonium chloride)-GO for the chronoamperometric sensing of prostate-specific antigen (PSA) level in blood at normal (i.e., <0.5 ng mL^−1^) or pathological level (i.e., >2.0 ng mL^−1^), yielding the detection limit and sensitivity of 10^−7^ ng mL^−1^ and 0.17 µA ng^−1^ mL, respectively. The CNT bundles were coated with PEDOT:PSS for the potentiometric sensing of Ca^2+^ ions with no significant interference by other non-target ions, yielding the detection range and sensitivity of 0.5–2.5 mM and ~4.0 mV mM^−1^, respectively. Lastly, the CNT bundles were coated with Pt nanoparticles, polyaniline, GOx, and Nafion for the chronoamperometric sensing of glucose level in blood vessels, yielding the detection range, sensitivity, and detection limit of 2.5–7.0 mM, ~5.6 nA µM^−1^, and 50 µM, respectively. For pH sensing, Wang et al. reported a stretchable form of electrochemical sensing e-textile using an elastomeric SEBS/Au fiber coated with polyaniline and an Ag/AgCl for the working and reference electrodes, respectively [111]. The e-textile exhibited a high sensitivity of 60.6 mV pH^−1^, linear pH detection range of 4–8, and stretchability up to a 30% tensile strain with little change (<2.6%) in the sensitivity.

Figure 4b shows an electrochemical sensing e-textile using a silk fabric-derived intrinsically nitrogen-doped carbon textile (SilkNCT) for the simultaneous detection of multiple biomarkers including glucose, lactate, ascorbic acid, uric acid, Na^+^, and K^+^ [112]. The electrical conductivity of the SilkNCT was adjusted by controlling its graphitization degree at a carbonization temperature ranging from 800 to 1050 °C. The highest electrochemical performance of the SilkNCT was achieved when carbonized at 900 °C. For glucose sensing, a mixture solution of GOx and chitosan was drop-casted onto the SilkNCT to form an amperometric sensor, yielding the sensitivity, linear detection range, and detection limit of 6.3 nA µM^−1^, 25–300 µM, and 5 µM, respectively. For lactate sensing, a mixture solution of oxidase and chitosan was poured onto the SilkNCT to form an amperometric sensor, yielding the sensitivity, linear detection range, and detection limit of 174 nA mM^−1^, 5–35 mM, and 0.5 mM, respectively. For Na^+^ and K^+^ ion sensing, the SilkNCT was coated with PEDOT:PSS and ion selective membranes and resulting sensors to form a potentiometric sensor, yielding the sensitivity, linear detection range, and detection limit of 51.8 and 31.8 mV, 5–100 and 1.25–40 mM, and 1.0 and 0.5 mM, respectively. For ascorbic and uric acid sensing, the pristine SilkNCT was used as a differential pulse voltammetry, yielding the sensitivity, linear detection range, and detection limit of 22.7 and 196.6 nA µM^−1^, 20–300 and 2.5–115 µM, and 1.0 and 0.1 µM, respectively. The e-textile was integrated with a portable circuit driver of signal collection and wireless data transmission, enabling the on-body, real time, and in situ analysis of the biomarkers.

Figure 4c shows an OECTs sensing e-textile using a cotton fiber coated with PEDOT:PSS as the channel, along with a Pt-gate electrode, for the detection of adrenaline regardless of saline contents in human physiological fluids [113]. In the electrolyte (i.e., sweat), adrenaline was electro-oxidized into adrenochrome at the surface of the Pt-gate electrode to generate protons across the source and gate terminals. The e-textile responded to a potential drop occurring at the interfaces of gate/electrolyte and electrolyte/PEDOT:PSS. Applying a positive gate voltage, cations from the electrolyte were moved toward the surface of PEDOT:PSS and de-doped by reducing PEDOT^+^ into PEDOT^0^, resulting in a decrease in the source-drain current due to the depletion of carriers. Without applying a gate voltage, the source-drain current increased. Notably, the overall response of the e-textile was reversible in a range of 1 × 10^−6^–1 × 10^−3^ M at a fixed gate-to-source voltage of 0.3 V, which was independent of the NaCl concentration of 0.01 and 0.1 M, respectively. 

Figure 4d shows an OECTs-sensing e-textile using a screen-printed PEDOT:PSS with the sheet resistance (*R*_s_) of 38.7 Ω sq^−1^ for both the channel and gate electrodes for the detection of various redox active molecules such as ascorbic acid, adrenaline, and dopamine [114]. With applying −0.9 and −0.3 V to the source-gate and source-drain electrodes, the electrochemical potential at the source and gate with respect to a saturated calomel electrode were 0.52 and −0.38 V, respectively. The electrochemical potential at the PEDOT:PSS channel (0.52 V > *E*_OX_ of analytes) was high enough to electro-catalyze the oxidation of biomolecules, resulting in the reduction of the channel conductivity due to the decreased number of positively charge carriers (i.e., PEDOT^+^). The e-textile exhibited a high performance in detecting redox active compounds including ascorbic acid (linear range = 10^−4^–10^−2^ M; sensitivity = 0.37 ± 0.03; and detection limit = 1 × 10^−5^ M), adrenaline (linear range = 3 × 10^−5^–5 × 10^−4^ M; sensitivity = 0.75 ± 0.07; and detection limit = 1 × 10^−5^ M), and dopamine (linear range = 2 × 10^−6^–3 × 10^−5^ M; sensitivity = 1.0 ± 0.2; and detection limit = 1 × 10^−6^ M). The operating voltage (<1 V) and absorbed power (~10^−4^ W) of the e-textile remained sufficiently low, making it suitable for portable and wearable applications.

Another notable example includes the e-textiles equipped with biofuel cells as an on-board power supply by harvesting biochemical energy from metabolites present in biofluids through electroenzymatic reaction, thereby enabling battery-free sensing [84,115,116]. Yin et al. demonstrated a biofuel cell using various compositions of screen-printable inks to textiles [84]. CNT-based pellets composed of enzymes, mediator, and electron transfer promoter were attached to interdigitate interconnections on textiles. Bioenergy was then harvested via the oxidation of lactate to pyruvate catalyzed by the lactate oxidase immobilized on an anode and the reduction of oxygen to water caused by bilirubin oxidase on a cathode, facilitating the detection of lactate up to a concentration of 25 mM. The biofuel cell was characterized using chronoamperometry with the maximum power of 21.5 µW per module when discharged at 0.5 V. For a conceptual demonstration, the biofuel cell was integrated with mechanical generator and successfully powered wearable liquid crystal display wristwatch and sensor-electrochromic display systems.

### 3.2. Gas Sensing

**Figure 5:**
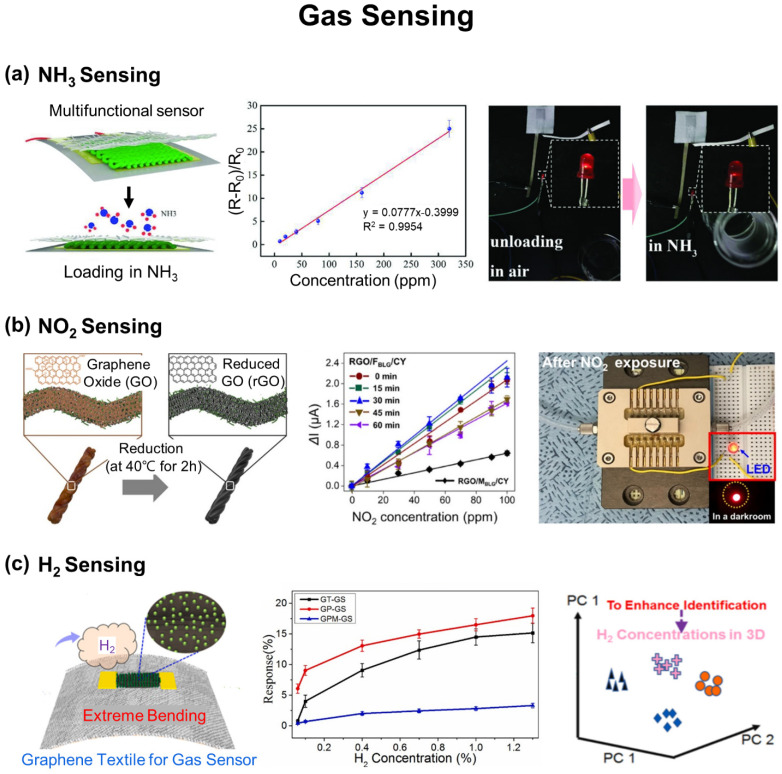
E-textiles for gas sensing. (**a**) NH_3_ sensing with the polyaniline@textile. Reprinted with permission from [104]. Copyright 2018 Wiley-VCH. (**b**) NO_2_ sensing with Amyloid/rGO yarns. Reprinted with permission from [117]. Copyright 2020 American Chemical Society. (**c**) H_2_ sensing with rGO/Pd NP-coated fabrics. Reprinted with permission from [94]. Copyright 2021 Elsevier B.V.

Figure 5a shows a gas sensing e-textile using fabrics coated with an in situ polymerized polyaniline and an interdigitated Cu/Ni polyester to serve as the top and bottom electrodes in detecting NH_3_, respectively [104]. The resistance of the e-textile increased and decreased linearly with and without exposing to NH_3_ in a range of 10–320 ppm, showing a little baseline drift (*R*^2^ = 0.9938) with a theoretical detection limit of ~92 ppb. The results indicated nearly reversible interaction between polyaniline and NH_3_. The e-textile also exhibited a high selectivity to NH_3_ among the other gases including ethanol, isopropanol, water, and acetone due to the unique protonation and deprotonation mechanism of polyaniline to NH_3_. The e-textile was also repeatable up to five cycles in recovering from its response to NH_3_ at a fixed concentration of 20 ppm.

Figure 5b shows a gas sensing e-textile for detecting NO_2_ using cotton yarns coated with rGO by amyloid nanofibrils as an adhesion promotor [117]. The rGO of the e-textile provided abundant active sites as an electron acceptor to adsorb NO_2_ molecules, resulting in a detectable increase in electrical potentials. The e-textile exhibited a high electrical conductance, response time, sensing efficiency, sensitivity, and limit of detection of 3.44 ± 0.37 µS, ~4 min, 0.52 ± 0.15 µA min^−1^, 25.93 ± 2.56 nA ppm^−1^, and 1 ppm, respectively. This high performance allowed the e-textile to alarm with a light-emitting diode for any higher concentration of NO_2_ beyond a threshold concentration (i.e., >10 ppm) that may cause harmful side effects on human health. 

Figure 5c shows a gas-sensing e-textile for detecting H_2_ using various fabrics coated with electron beam (e-beam) evaporated Pd nanoparticles and inkjet-printed rGO [94]. The resistance of the e-textile changed by the adsorption and desorption of H_2_ to/from the Pd NPs on the rGO. The e-textile exhibited about six-times higher performance in detecting H_2_ comparing to conventional gas sensors built on a polyimide substrate in a concentration range of 1000 ppm to 1% due to the substantially increased surface area of the fabrics. The e-textile was also integrated with a triboelectric unit to power the Bluetooth module. To further increase the sensing accuracy, the initial resistance was calibrated through a machine leaning-enabled data processing to rule out the effect of surrounding temperature and humidity on sensing. 

## 4. Electrophysiological Sensing

Electrophysiological signals, such as electrocardiogram (ECG), electromyogram (EMG), and electroencephalogram (EEG), are the result of measurable biopotential differences by the polarization of ion channels in the cell membrane during energy consumption (i.e., cell polarization). The electrophysiological signals can be captured on the skin in a non-invasive manner to provide useful information in many applications such as routine health monitoring, rehabilitation after injuries, prosthetics, and brain–computer interfaces. For instance, ECG signals represent the electrical activity of the heart including cardiac rhythms and ischemic changes [118,119,120,121] The analysis of main peaks (i.e., P-QRS-T complex) in ECG signals is useful in diagnosing heart diseases such as heart conduction disorders and arrhythmias. The lack of P-waves in ECG signals may indicate atrial fibrillation or stroke. The variation in R-R intervals in ECG signals is a sign of sleep apnea. Therefore, the survival rate and treatment efficacy of patients with a heart disease could be increased from continuously monitoring ECG signals. Electrodes made of PEDOT:PSS [122,123,124,125,126], CNT [127], graphene [128], Ag NPs [125,129,130,131,132,133], Ag flakes [134], and Ag NWs [135] have been typically employed in e-textiles for the reliable collection of ECG signals. EMG signals represent a myoelectric potential activity of skeletal muscles, displaying the health conditions of muscular cells [136,137]. Analyzing the pattern of EMG signals is useful in evaluating muscular injuries or their rehabilitation process. EMG signals are relatively localized on target muscles with the typical signal amplitude of 0–10 mV at the filter setting of 1 Hz–1 kHz. Metallic electrodes using Au film [138], Ag flakes [31,134,139], or Ag NWs [135] have been typically employed in e-textiles for the reliable collection of EMG signals. EEG signals represent the electrical activity of the brain, which are useful in assessing epileptic event, unresponsive wakefulness state, minimally conscious state, and even coma state [140,141]. For instance, alpha and beta waves in EEG signals indicate blinking and active thinking, respectively. However, the long-term acquisition of EEG signals remains relatively challenging by hairs, which requires a high air-water permeability of recording electrodes. Electrodes made of highly conductive silver-based materials [32,142,143,144] or a few alternatives including conductive polymers [145] and graphene derivatives [146] have been typically employed in e-textiles for the reliable collection of EEG signals. 

Electrophysiological sensing electrodes collect electrical currents by transducing ionic current through the skin, which are classified as polarizable or non-polarizable electrodes [119]. The non-polarizable electrodes (e.g., Ag/AgCl electrodes) are mostly employed for collecting electrophysiological signals because they can pass actual current between the skin and the electrode. The measurement of skin impedance is relied on electrochemical impedance spectroscopy wherein the skin-electrode contact impedance can be reduced to improve signal quality. The conductivity of the skin increases from dry (10^−7^ S m^−1^) to wet condition (10^−5^ S m^−1^) [147]. Hence, an electrolyte gel is generally used. However, the gelled electrodes not only dry out over an extended recording time but also may bring about skin irritation. To tackle this issue, dry electrodes in e-textiles have been investigated without compromising their SNR [127,128,148,149]. Furthermore, additional coating of Au nanoparticles over the Ag NPs has been also explored to improve the biocompatibility by preventing a direct contact of Ag to the skin [150].

In this section, a number of e-textiles are reviewed for the high-fidelity monitoring of electrophysiological signals in contact with the skin.

### 4.1. ECG Sensing

**Figure 6:**
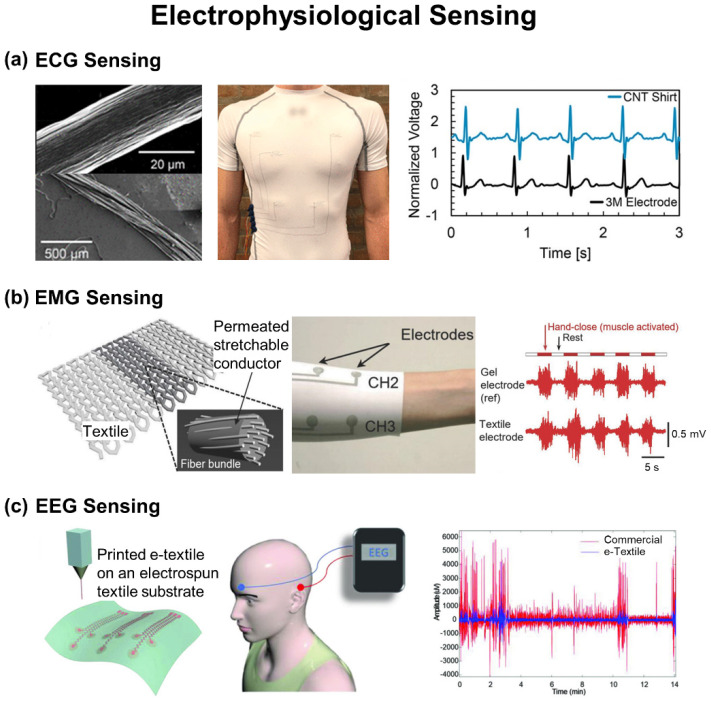
E-textiles for electrophysiological sensing. (**a**) ECG sensing with solution-spun CNT threads. Reprinted with permission from [127]. Copyright 2021 American Chemical Society. (**b**) EMG sensing with Ag flake-printed fabrics. Reprinted with permission from [31]. Copyright 2017 Wiley-VCH. (**c**) EEG sensing with Ag particle/fluoropolymer composite ink-printed electrospun fabrics. Reprinted with permission from [143]. Copyright 2018 Wiley-VCH.

Figure 6a shows an ECG-sensing e-textile made of a stretchable and sewable conductive thread that was formed through a wet-spinning of CNT fibers [127]. The CNT fibers exhibited an average diameter, aspect ratio, and length of ~1.8 nm, ~4100, and ~7.4 µm, respectively. Specifically, the thread was formed by piling the CNT fibers using a custom rope-making machine with the final diameter and density of 22.0 ± 1.0 µm and 1580 ± 80 kg m^−3^, respectively. The thread was mechanically robust with a tensile strength of 1.7 ± 0.2 GPa, making it applicable to a standard sewing machine and laundry machine without compromising its electrical conductivity of 6.6 ± 0.7 MS m^−1^. The thread was stitched in a shirt into an array of recording electrodes for ECG sensing, demonstrating a high SNR comparable to that of commercial 3M electrodes regardless of subjects (*p* > 0.05). Negligible degradation in SNR was observed after 1000 cycles of stretching at the applied strain of 60%.

As another notable example, Yapici and Alkhidir reported a dip-coating/thermal cladding method to prepare a conductive graphene-clad nylon (6 cm × 3 cm) in an elastic band [128]. The resulting recording electrodes were then dipped into a GO solution, followed by a thermal treatment and dipping in a hydrogen iodide to reduce the GO into graphene to further increase the electrical conductivity. Two types of e-textiles were demonstrated in the form of a wristband and neckband for ECG sensing. The wristband contains two pairs of recording electrodes on both sides with one reference electrode on one side. In contrast, the neckband contains all the electrodes on the same side in the middle. For wireless monitoring, both the wristband and neckband were connected to a lithium-ion polymer battery (3.7 V and 2000 mAh) and a wireless communication module (BlueSMiRF Silver). The wristband was capable of measuring ECG signals with a higher SNR than the neckband due to a large biopotential difference between reciprocal electrodes. The ECG signals obtained from the wristband were comparable to those of using conventional Ag/AgCl electrodes, even without the use of an ionic conductive gel.

### 4.2. EMG Sensing

Figure 6b shows an EMG sensing e-textile using an Ag ink that provides a high stretchability (up to 450%) and recovery (under 100%) [31]. The Ag ink was comprised of fluoroelastomer, butyl carbitol acetate (BCA), and Ag flakes with an average size of 2–3 µm, in which its viscosity and permeability were controllable to be easily printable through a nozzle. The fluoropolymer provided the softness and stretchability to the composite. Because the BCA has a higher boiling point (247 °C) and low vapor pressure (<0.01 mmHg) than acetone, the Ag ink was highly permeable into fabrics. This aspect allowed for multiple passes of printing to substantially decrease the resistance to 26 and 3 Ω at stretched and released state after 1000 cycles, respectively. The resulting e-textile was demonstrated in a form of a skin-tight compression sleeve for EMG sensing, exhibiting a sheet resistance of 0.06 Ω sq^−1^ which increased up to 70 times under stretching at the applied strain of 450%. A thin layer of thermoplastic polyurethane (TPU) was used as an encapsulation to prevent the e-textile from sweat, abrasion damage, and redundant skin contact. To further enhance the cyclic stability and long-term durability, highly durable nanofiber-reinforced elastic Ag flakes can be also incorporated [134]. For instance, randomly aligned long poly(vinylidene fluoride) nanofibers were added in Ag flakes, which resulted in an outstanding initial conductivity of 9903 S cm^−1^, sheet resistance of 0.047 Ω sq^−1^, and stretchability up to 800% with a low cyclic degradation (Δ*R*/*R*_0_ = 0.56 after 5000 cycles of stretching at the applied strain of 50%). The resulting e-textile was stretchable up to 30% on the skin and over 100% at joints without a noticeable degradation in performance. 

Yao et al. invented a laser scribing/heat press lamination process to transfer conductive Ag NWs to fabrics [135]. Specifically, Ag NWs were coated on a glass using a Meyer rod with a TPU solution as an encapsulation layer to form an embedded structure of Ag NW/TPU. The Ag NW/TPU layer was pattered at a high resolution (up to 135 µm in line width) with a laser cutter and then transferred onto fabrics, followed by heat pressing. The resulting e-textile was stretchable, foldable, twistable, and washable without compromising their electrical conductivity and density of 5030 S m^−1^ and 0.48 mg cm^−2^, respectively. More recently, Chang et al. utilized a programmable dual regime spray system, enabling the direct custom writing of conductive nanoparticles into fabrics at high resolution (submillimeter in line width) over large areas (up to meters scale) [151]. An array of recording electrodes with a high conductivity (9400 S cm^−1^) was sprayed into stretch horse blankets across a large surface (1.3 m^2^) with negligibly compromising the intrinsic fabric properties of wearability. No substantial change in electrical conductivity was observed up to the applied strain of 50%. The resulting e-textile was applied to the remote monitoring of ECG, EMG, and abdominal strains from the chest, forelimb shoulder, and abdomen of horses under ambulatory conditions.

### 4.3. EEG Sensing

Figure 6c shows an EEG sensing e-textile formed through a direct writing of a conductive Ag NPs/fluoropolymer composite ink into electrospun fabrics [143]. The resulting recording electrodes exhibited a conductivity of 3200 S cm^−1^, which degraded after 1000 cycles at the applied strain of 30%. For measurement, the recording electrodes were placed on the skin behind the ear to capture EEG signals with less motion artifacts comparing to commercial gel electrodes. To minimize the change in impedance at the interface between the skin and electrodes on the irregular scalp surface, Muthukumara et al. suggested the use of polyaniline-coated PU foam electrodes in cotton through the in-situ polymerization of aniline [145]. The polyaniline-coated PU foam electrodes were soft and compressive up to 8.69% at 0.7 N cm^−2^, while exhibiting an impedance of 1.45 MΩ with a sheet resistance of 7 kΩ sq^−1^ when measured at a relative humidity of 65%. Shu et al. also introduced a multilayered porous foam of recording electrodes in fabrics, in which the contact impedance of electrode-scalp (8.97 kΩ at 10 Hz) was lower than that of conventional wet electrodes with a conductive gel (11 kΩ at 10 Hz) [144]. The porous foam was able to quickly absorb sweat, which could decrease the impedance down to 4.04 ± 1.97 kΩ at 10 Hz. The porous foam also provided a long-term stability in EEG sensing with a small impedance variation of 5 kΩ in 8 h, which is greatly favorable over conventional recording electrodes with a conductive gel that dries out over time.

## 5. Wearable Displays

Wearable displays that can deform and fit the contour of the human body have been of great interest for visual expression and communications in real time [152]. To this end, a number of e-textiles have been developed by utilizing either direct current (DC)-driven LEDs or alternating current (AC)-driven ACELs [11,21]. The DC-driven LEDs are typically comprised of various functional layers, including hole transport, light emitting, and electron transport layers, that are sandwiched between an anode and cathode. They include PLECs [153,154], OLEDs [155,156,157,158,159,160,161], phosphorescent organic light-emitting diodes (phOLEDs) [162,163], and PLEDs [164,165]. They can be integrated directly into garments and fabrics, but their application remains impeded by the requirement of complicated and expensive fabrication processes. The AC-driven ACELs are typically configured into coaxial fibers [166,167,168,169] or multilayered fabrics [170,171,172,173] comprised of an emissive layer as well as inner and outer electrodes. For an instance, the simplest version of the AC-driven ACELs is comprised of impurity-doped zinc sulfide (ZnS) phosphor dispersed in a dielectric elastomer that is sandwiched between top and bottom transparent conductive electrodes [174]. Unlike the DC-driven LEDs, they are relatively simple to fabricate without the need for stacking delicate and complex layers, but their application remains impeded by low luminance and high operating voltage [175].

In parallel, colorimetric displays have been also demonstrated by coating fabrics with either electrochromic (e.g., PEDOT [37,176], polyaniline [177], WO_3_ [178], poly(3-methylthiophene) [179], viologens [180]) or thermochromic (e.g., pigment dyes [38,181,182]) materials. The electrochromic materials are sandwiched between transparent electrodes and gel electrolytes, which can change their optical property according to different visible region absorption bands during redox reactions with an applied electrical potential across the electrodes. The electro-thermochromic devices have been demonstrated through the combination of a thermochromic layer and Joule heating fabrics. The color of thermochromic layer can be changed when heated by the Joule heating fabrics above the activation temperature. In the following sections, a number of e-textiles are reviewed in their use for wearable display.

### 5.1. DC-Driven LEDs 

**Figure 7:**
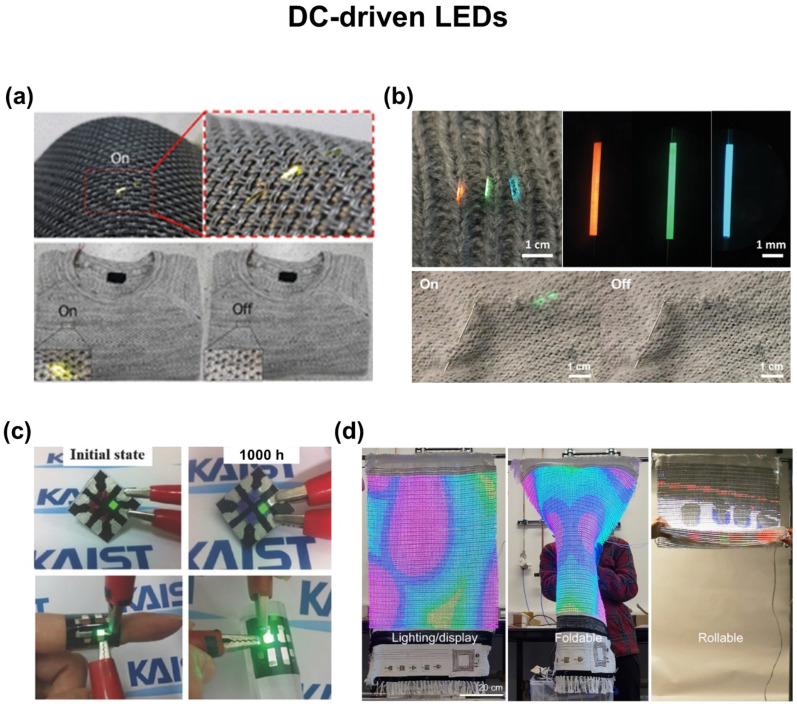
E-textiles for DC-driven LEDs. (**a**) Fiber-shaped OLEDs hand-woven into garments. Reprinted with permission from [183]. Copyright 2017 American Chemical Society. (**b**) Fiber-shaped multicolor phOLEDs woven into daily clothes. Reprinted with permission from [162]. Copyright 2021 Wiley-VCH. (**c**) Fabric-based OLED based on the PU planarization layer and multilayers of barrier/capping films. Reprinted with permission from [184]. Copyright 2016 Wiley-VCH. (**d**) Fabric-based large-scale RGB-colored LEDs. Reprinted with permission from [185]. Copyright 2022 Springer Nature.

Figure 7a shows an e-textile for a wearable display with DC-driven LEDs with a comparable luminescence efficiency to conventional OLEDs built on a glass [183]. The e-textile was formed by subsequently depositing PEDOT:PSS, zinc oxide nanoparticles (ZnO NPs)/polyethylenimine (PEI), Super Yellow, molybdenum oxide (MoO_3_), and Al on a 300 µm-think polyethylene terephthalate (PET) fiber to act as the cathode, electron injection layer, emissive layer, hole injection layer, and anode, respectively. The entire deposition process was implemented by dip-coating and thermal annealing, except for the anode deposition that was carried by thermal evaporation under a vacuum condition. The e-textile exhibited a high luminance (>10,000 cd m^−2^) and efficiency (>11 cd A^−1^) with a long operating lifetime (>80 h). In addition, the e-textile endured tensile strain up to 4.3% at a radius of curvature of 3.5 mm without a noticeable degradation in performance.

Figure 7b shows an e-textile for wearable display with phOLEDs having higher internal efficiencies and lower driving voltages comparing to those of OLEDs [162]. The e-textile was configured into an inverted structure with PEDOT:PSS, ZnO NPs/PEI, RGB phosphorescent emissive layers on the basis of poly(*N*-vinyl carbazole), 4,4′,4″-tris(carbazol-9-yl)-triphenylamine, MoO_3_, and Al that were deposited on a 300 µm-think PET fiber for use as the cathode, electron injection layer, emissive layer, hole transport layer/exciton blocking layer, hole injection layer, and anode, respectively. A 50 nm-thick Al_2_O_3_ encapsulation layer was deposited using an atomic layer deposition to prevent oxidation from moisture and oxygen. The e-textile exhibited the highest current efficiencies of 16.3, 60.7, and 16.9 cd A^−1^ at a driving voltage of <8 V with the luminance of 4462, 11,482, and 1199 cd m^−2^ for red, green, and blue, respectively. Furthermore, a matrix-designed phOLED display was also demonstrated by consisting of four conductive scan lines and four luminescent data lines. To ensure contact between the scan and luminescent data lines, a 100 µm-thick PET fiber was used to fasten the emitting unit cell. The e-textile was capable of visualizing letters of information that were wirelessly received from a computer via a microcontroller board (Arduino Uno).

Figure 7c shows an e-textile for wearable display with using an inverted top-emitting OLED that was constructed on a PU planarization layer and multilayers of barrier/capping films on a rough fabric [184]. A 20–40 µm-thick PU layer was coated on a rough fabric to flatten the surface prior to the fabrication of OLEDs. The OLEDs were stacked with multiple layers including 100 nm-thick Al, 2 nm-thick 8-hydroxyquinolatolithium, 50 nm-thick tris(8-hydroxy-quinolinato) aluminum, 50 nm-thick *N,N′*-bis(naphtanlen-1-yl)-*N,N′*-bis(phenyl)-benzidine/zinc sulfide (NPB), and 5 nm-thick WO_3_ to act as the cathode, electron injection layer, emission layer, hole transport layer, and hole injection layer, respectively. The NPB layer was capped by layers of 30 nm-thick ZnS (30 nm) to prevent contamination. Additional encapsulating layers of ~30 nm-thick aluminum oxide and ~300 nm-thick poly(vinyl alcohol) were added on the bottom and top of the OLEDs to act as a planar surface and oxidation-free layer, respectively. The resulting e-textile exhibited a stable performance with only a few dark spots under operation up to 3500 h in an ambient environment. The e-textile was mechanically robust with little change in performance against 1000 cycles of bending at a bending radius of 20 mm.

Figure 7d shows an e-textile for wearable display by utilizing 120 × 65 pixels of RGB LEDs over a large area (34 inches) that contains a total of 23,400 sub-pixels on cotton [185]. The e-textile was fabricated by a modified conventional LED pick-and-place method on Cu fibers with a predesigned pixel-to-pixel pitch of 7 mm and 5 mm for low- and high-resolution LEDs, respectively. The brightness of RGB colors exceeded 10,000 cd m^−2^ while maintaining their performance under mechanical deformations such as folding, bending, and rolling. The e-textile was also integrated with six input units including radio frequency antenna, photodetector, touch sensor, temperature sensor, biosensor, and energy storage over a large area (46-inch), allowing for the wireless real-time monitoring of distance between RF antenna and transmitter (0–6 cm at a frequency of 13.56 MHz), UV irradiation (0.5 mW), touch duration by hand (1–30 s), temperature of finger (5–70 °C), ECG (voltage gain at ~28 *V/V*), and power supply (total output voltage at 5 V), respectively.

### 5.2. AC-Driven ACELs

**Figure 8:**
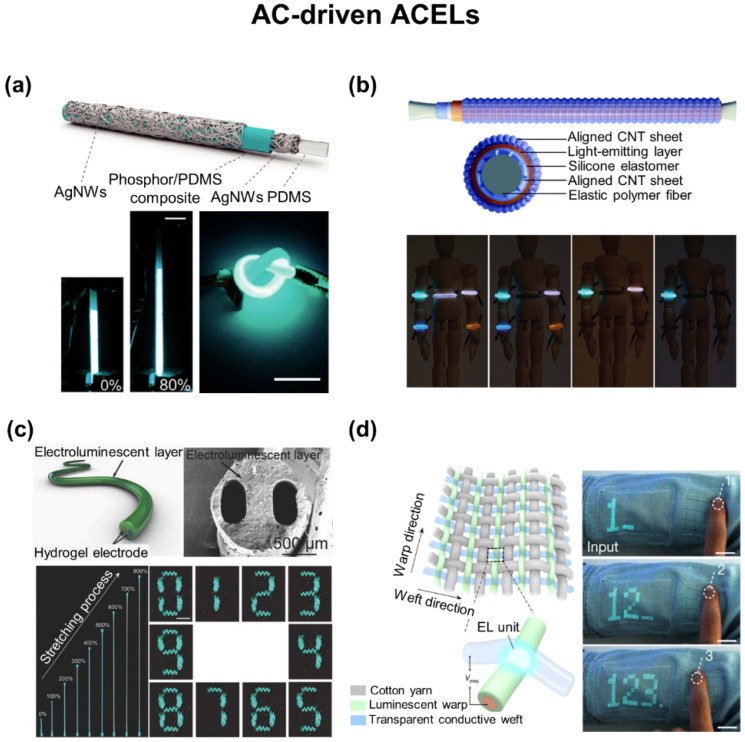
E-textiles for AC-driven ACELs. (**a**) Stretchable ACEL fiber with Ag NW electrodes. Reprinted with permission from [186]. Copyright 2018 MDPI. (**b**) Color-programmable ACEL fiber with aligned CNT sheets. Reprinted with permission from [187]. Copyright 2018 Royal Society of Chemistry. (**c**) Stretchable ACEL fiber with ionic hydrogel electrodes. Reprinted with permission from [188]. Copyright 2018 Wiley-VCH. (**d**) ACEL fabric with transparent conducting wefts and luminescent warps. Reprinted with permission from [36]. Copyright 2021 Springer Nature.

Figure 8a shows an ACEL fiber comprised of ~100 nm-thick Ag NWs and ~55 µm-thick ZnS phosphor on a 2.5 mm-thick elastic PDMS core to act as the inner/outer electrodes and emissive layer, respectively [186]. Firstly, the PDMS core was prepared by curing a PDMS prepolymer in a template of plastic tube with a diameter of 2.5 mm. After removing the template, a solution of Ag NWs was spray-coated onto the PDMS fiber while simultaneously rotating and heating to form a homogeneous layer. An emissive layer of ZnS phosphor/PDMS was then dip-casted onto the Ag NW-coated PDMS fiber. Finally, a solution of Ag NWs was spray-coated again as an outer electrode. The resulting ACEL fiber exhibited the maximum luminance of 307.3 cd m^−2^ at a frequency and voltage of 5 kHz and 500 V, respectively. This ACEL fiber was also stretchable up to 80% and reliable against 6000 cycles of stretching at the applied strain of 50%.

Figure 8b shows a color-programmable ACEL fiber consisting of an Ecoflex, CNT sheet, and ZnS phosphor/Ecoflex composite layer to act as the core fiber, inner/outer electrodes, and emissive layer, respectively [187]. The CNT sheet was continuously wrapped around a pre-stretched core fiber at an angle of 45° using a motor to form uniform inner electrodes. Then, a light-emitting tube made of the pre-stretched ZnS phosphor/Ecoflex composite in a thickness of 350–400 µm was separately wrapped with the CNT sheet. The light-emitting tube was then integrated with the inner electrodes to complete the fabrication. The resulting ACEL fiber exhibited the maximum luminance of 12.66 cd m^−2^ at a frequency and voltage of 1.5 kHz and 6 V µm^−1^, respectively. The ACEL fiber was also stretchable up to 200% and reliable against 200 cycles of stretching at the applied strain of 200% with a negligible change (<2.5%) in the emission intensity. 

Figure 8c shows an ACEL fiber with two inner parallel ionic hydrogel electrodes on the basis of poly(vinyl alcohol), poly(ethylene oxide), and an outer emission layer with a mixture of ZnS phosphor/Dragon skin fabricated via a continuous one-step extruding [188]. The ionic hydrogel electrodes were mechanically stretchable up to 800%, optically transparent with a transmittance over 92%, and electrically conductive with an ionic conductivity of ~0.29 S cm^−1^ at a wavelength range of 400–850 nm. The ACEL fiber exhibited the maximum luminance of 233.4 cd m^−2^ at a frequency and voltage of 1.5 kH and 7.7 V µm^−1^, respectively. The ACEL fiber was also mechanically robust without a noticeable degradation in performance against 100 cycles of stretching at the applied strain of 300%. This feature allowed the ACEL fiber to be directly woven into clothes in an array to display a seven-digit number. However, the lamination of the ACEL fibers on fabrics inevitably reduces the breathability of the resulting e-textiles. In addition, fine patterning in pixilation is limited because the conductive and emissive materials are highly diffusive on fabrics during their repetitive screen-printing processes. 

Figure 8d shows a 6 m-long and 25 cm-wide e-textile containing 5 × 10^5^ ACEL units with an 800 µm-spacing in a knitted configuration of transparent conducting wefts and luminescent fiber warps [36]. The uniform luminescent fiber warp was prepared by dip-coating a ZnS phosphor/TPU slurry on a silver-plated conductive yarn. For the transparent conducting weft fiber, an ionic-liquid-doped TPU was melt-spun via a nozzle with a diameter of 0.25 mm. The ACEL units were illuminated at every contact point between wefts and warps, exhibiting a luminance of 115.1 cd m^−2^ at a voltage and frequency of 3.7 V µm^−1^ and 2 kHz, respectively. The current density and power consumption of the e-textile were 1.8 mA cm^−2^ and 363.1 µW, respectively. The e-textile provided a highly uniform brightness with a deviation less than 8%, and also remained stable after bending, stretching, and pressing. For demonstration, a 4 × 4 keyboard was fabricated in a fabric by weaving four low-resistance warps (Ag-plated yarns) with four high-resistance wefts (carbon fibers), where the intersections of warps and wefts are to detect a touch. As a power source, photoanode wefts were added by depositing a photoactive layer of titanium dioxide nanotubes on a titanium wire to form an array of solar cells. Additionally, a battery was used consisting of manganese dioxide-coated CNT fiber, zinc wire, and zinc sulfate gel to act as the cathode, anode, and electrolyte, respectively. The fully integrated system, along with a Bluetooth unit, was used to display the wearer’s location, input information (e.g., number, message), and mental states (e.g., relaxed, anxious) associated with EEG signals.

### 5.3. Colorimetric Display

**Figure 9:**
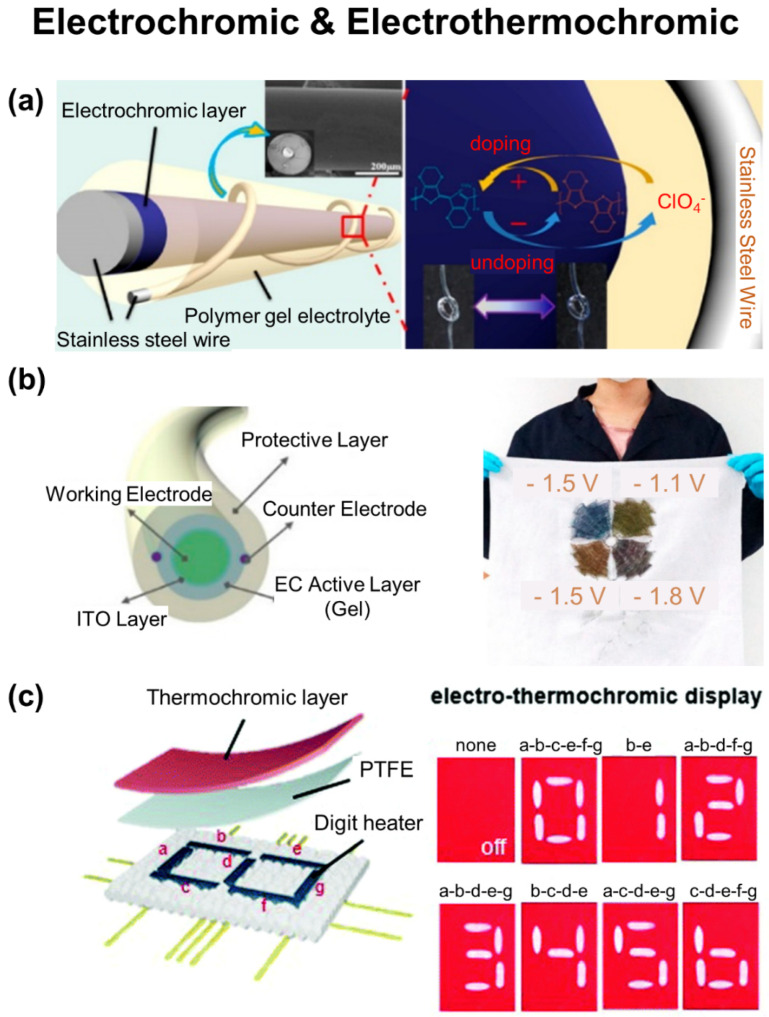
E-textiles for electrochromic and electrothermochromic display. (**a**) RGB-colored electrochromic fiber based on PEDOT and stainless-steel wire. Reprinted with permission from [179]. Copyright 2014 American Chemical Society. (**b**) Multicolor electrochromic fiber based on dual-counter-electrodes of Cu@Ni wires. Reprinted with permission from [180]. Copyright 2020 American Chemical Society. (**c**) Electrothermochromic textile based on the PEDOT:PSS/Ag NWs-based Joule heater and thermochromic layer. Reprinted with permission from [181]. Copyright 2019 Royal Society of Chemistry.

Figure 9a shows an RGB-colored electrochromic fiber that contains different kinds of electrochromic *π*-conjugated organic polymers, such as PEDOT, poly(3-methylthiophene), and poly(2,5-dimethoxyaniline), on a stainless-steel wire [179]. The electrochromic fiber was then coated with a polymer gel electrolyte on the basis of lithium perchlorate in propylene carbonate and 25 wt% polymethyl methacrylate and then twisted by another stainless-steel wire. The resulting electrochromic fiber exhibited redox peaks at 1.2 V and −2.5 V with the corresponding response time of 0.128 and 0.104 s for coloring and bleaching, respectively. The electrochromic fiber was integrated into the warp and weft of a black nylon fabric to display patterns of words by changing color between dark blue and gray, red and black, and dark yellow and gray at the applied voltage of −2.6 V and 1.6 V, −2 V and 2 V, and −1.4 V and 1.4 V, respectively.

Figure 9b shows a hundreds-of-meters long multicolor electrochromic fiber having parallel dual-counter-electrodes of Cu@Ni wires that were sequentially coated with a ~0.8 µm-thick indium tin oxide (ITO), ~0.12 mm-thick electrochromic viologen layer, and ~0.20 mm-thick polymer protective layer [180]. By introducing different kinds of viologens, the electrochromic fiber displayed multiple colors including blue, magenta, green, and dull red. The electrochromic fiber was able to be woven into nylon yarns to change color from gray, yellow, and gray to magenta, green, and blue at the applied voltage of −1.7 V, −0.9 V, and −1.2 V, respectively. The resulting e-textile was highly air permeable, thermal and moisture resistive, and durable.

Figure 9c shows an electrothermochromic e-textile containing a stencil-printed circuit on cotton [181]. The surface of the cotton was treated with (3-aminopropyl) trimethoxysilane to promote adhesion. A flexible Joule heater was constructed with a layer-by-layer mixture of PEDOT:PSS/Ag NWs and a chitosan solution, followed by the deposition of a protective layer (e.g., polytetrafluoroethylene) with a spray-coater. The Joule heater exhibited an electrical resistance of 46 Ω and was intact against various deformations (e.g., bending, stretching, heating) at 85 °C and wetting at 85 RH%. The resulting e-textile was able to display seven-digit patterns of numbers from 0 to 9 by selectively heating the segments.

## 6. Conclusions and Perspective

Herein, we review the current progress and challenges of e-textiles in wearable sensing and display with a focus on their key materials, working principles, fabrication methods, and potential applications. E-textiles in response to temperature, strain, and pressure have been applied to track skin temperature and body motions with rapid response time. E-textiles in response to chemical stimuli have been applied to detect biomarkers, biomolecules, and metabolites from body fluids for tracking pathophysiologic conditions associated with chronic diseases such as diabetes, stroke, gout, myocardial infarction, and Parkinson’s disease. E-textiles in response to external gas have been applied to convey immediate information to the wearer under invisible and dangerous environmental circumstances. E-textiles in response to electrophysiological responses from the skin have been applied to continuously record vital signals for the early detection of abnormal symptoms. Some of the e-textiles can be potentially integrated with a closed-loop display to visually transmit the sensing data or other immediate information to the wearer for real-time communications. DC- and AC-driven electroluminescence LEDs have been incorporated into e-textiles with tunable brightness and color. DC offers a higher efficiency in luminance and color characteristics over AC, but requires further improvement in mechanical flexibility, power consumption, and production cost for active-matrix arrays. Alternatively, colorimetric dyes have been also incorporated into e-textiles through a relatively simple patterning process for binary on/off data output with a low power consumption.

In addition to the abovementioned sensors based on electrically conductive electrodes, optical fiber sensors are one of the promising candidates not only in the field of wearable health monitoring, such as gait analysis, evaluation of instrumented insoles, physiological parameters monitoring, robotic devices for rehabilitation, and biosensors for pathologies detection. These sensors offer unique advantages of zero sensitivity drift, high accuracy, high strain limits, high sensitivity, and insensitivity to electromagnetic interference. Recently, polymeric optical fibers have been developed with outstanding mechanical flexibility, high breaking strain limits, and biocompatibility compared to conventional silica-based glass optical fibers. Despite great progress, optical fiber sensors still have a limitation in securing reliability, where light coupling can cause instability in the long-term monitoring of applications that typically require a solid and stable connection to data acquisition equipment. The requirement of self-calibration is also a concern in wearable sensing applications due to the large variability of the optical properties. The polymeric optical fibers are still not flexible enough to be integrated with e-textiles.

Despite tremendous advances, several challenges remain in implementing e-textiles at a commercial scale while maintaining their mechanical integrity. For instance, e-textiles may degrade in their mechanical integrity over time due to the potential delamination or damage of the coating materials on fabrics against multiple uses and laundry cycles, or even extreme user conditions such as excessive stretching, twisting, and heating. A number of polymers, such as parylene, polyurethane, polydimethylsiloxane, and polyimide, have been explored for encapsulating e-textiles, but their waterproof effect is limited due to a high water-vapor transmission rate (WVTR). Ceramic coating offers a relatively low WVTR to protect e-textiles from moisture over a significantly expected time (i.e., years), but its utility is impeded by the requirement of a complicated, expensive, and time-consuming process. Therefore, further improvements are needed for effectively encapsulating e-textiles in both adhesion and waterproof qualities, not only to maintain their mechanical integrity but also to prevent them from oxidation, abrasion, or electric shock in daily use or multiple laundry cycles. Alternatively, it might be also worth considering the development of low-cost and disposable e-textiles, which can thereby eliminate the requirement of cleaning, disinfection, or sterilization after each use. 

Scalable production of e-textiles up to meter scale without compromising their intrinsic properties and performances is also critical. Several strategies by utilizing a continuous solution-extrusion have been employed in producing e-textiles over meter scale. Nonetheless, most of the current manufacturing processes are limited to a lab-scale fabrication and rely on the use of expensive and environmentally unfriendly equipment. Thus, a successful technology transfer of lab-scale prototyping to industrial scale production is a potential direction.

Despite great progress, many challenges still remain to be addressed in terms of the production method, design layout, and implementation of e-textiles. The quantification of analysis should also be optimized in electrochemical sensing via a thorough calibration of data. In addition, the stability of e-textiles in chemical sensing should be secured against biofouling on the surface of electrodes, which possibly results in incorrect sensing responses. This issue may be further deteriorated when biorecognition elements, such as peptides, antibodies, or enzymes, are used. While the detection limit of e-textiles has reached a satisfactory level to date, their repeatability and reusability need further improvement. The sample collection in e-textiles also need further improvement without compromising intrinsic fabric characteristics. Moreover, another key challenging issue remains in the unobtrusive connection of a power source to e-textiles. 

Securing the biosafety of e-textiles is also a critical consideration to eliminate any risk of inflammatory or toxic responses to the skin. To this end, biocompatible metals (e.g., Ag, Au, Pt, Ti) with a high corrosion resistivity have been employed for conductive elements, but they are typically expensive. Alternatively, carbon-based nanomaterials (e.g., graphene derivatives, carbon nanotubes) or conductive polymers (e.g., polypyrrole, polyaniline, poly(3,4-ethylenedioxythiophene):poly(styrene sulfonate)) have been employed, but their long-term biosafety remains controversial due to the use of toxic solvents or surfactants during their synthesis. These materials may require an additional coating of medical-grade encapsulation (e.g., SEBS, SBS, PDMS). Moreover, the operating voltage of e-textiles for wearable display should remain below the shock hazard threshold voltage (i.e., 50 V_rms_) while retaining a sufficient level of luminance.

The e-textiles in chemical sensing can also benefit from integrating with a wireless communication module to monitor and transfer the wearers’ clinical data for self-diagnosis and protection. Many wireless communication modules, such as near-field communication (NFC), radiofrequency identification (RFID), Bluetooth, and ZigBee, have been utilized in several wearable devices for fast data transmission. When choosing an appropriate wireless communication module, several important criteria should be carefully considered, including the transmission range, data rate, power consumption, and bandwidth. An NFC is a near-field wireless communication system with low power (~15 mW) and even operates without a battery in a passive mode, but with a short working distance (5–10 cm). A RFID, which is called an electronic tag, identifies information using radiofrequency. Passive RFID in particular has been widely adopted as a data transmission method for wireless sensors with a wide operating frequency (i.e., 120–140 kHz, 13.56 MHz, and 868–956 MHz for low-, high-, and ultrahigh-frequency identification, respectively). However, its signal range is relatively short and complicated to process. Contrary to a classic Bluetooth consuming higher power than other wireless communication modules, a Bluetooth low energy provides low-power wireless connection (~1 mW) with an enough communication range (up to 10 m) for wireless personal area network (WPAN) communication. In addition, a ZigBee has an advantage of being relatively simpler and cheaper than WPAN with a wireless communication over a wide distance (up to 100 m) and low power consumption (~50 mW). However, a ZigBee has a low data rate (~250 kb s^−1^). These modules, due to their rigid formfactor, are currently challenged to be monolithically integrated with textiles, which restricts the fabrication of all-in-one wireless e-textiles. Therefore, further development of efficient wearable antennas, energy-efficient wireless communication tools, and their monolithic integration into e-textiles is demanding.

In summary, the recent advances in e-textiles have paved the way in both wearable sensing and display. Further advances in their production at an industrial scale and in a cost-effective manner will accelerate the translation of e-textiles into practice. Diversifying the encapsulation materials across arbitrary fabrics will increase the versatility of e-textiles to be intrinsically stretchable, adhesive, waterproof, and transparent in wearable sensing and display.

## Data Availability

The data presented in this study are available on request from the corresponding author.
